# The current status and future of PD-L1 in liver cancer

**DOI:** 10.3389/fimmu.2023.1323581

**Published:** 2023-12-12

**Authors:** Liyuan Hao, Shenghao Li, Jiali Deng, Na Li, Fei Yu, Zhi Jiang, Junli Zhang, Xinli Shi, Xiaoyu Hu

**Affiliations:** ^1^ School of Clinical Medicine, Chengdu University of Traditional Chinese Medicine, Chengdu, Sichuan, China; ^2^ Department of Infectious Diseases, Hospital of Chengdu University of Traditional Chinese Medicine, Chengdu, Sichuan, China; ^3^ Clinical Research Center, Shijiazhuang Fifth Hospital, Shijiazhuang, Hebei, China; ^4^ Department of Infectious Diseases, Jiangsu Province Hospital of Chinese Medicine, Nanjing, Jiangsu, China; ^5^ Center of Experimental Management, Shanxi University of Chinese Medicine, Jinzhong, China

**Keywords:** PD-L1, hepatocellular carcinoma, liver cancer, cancer, HCC

## Abstract

The application of immunotherapy in tumor, especially immune checkpoint inhibitors (ICIs), has played an important role in the treatment of advanced unresectable liver cancer. However, the efficacy of ICIs varies greatly among different patients, which has aroused people’s attention to the regulatory mechanism of programmed death ligand-1 (PD-L1) in the immune escape of liver cancer. PD-L1 is regulated by multiple levels and signaling pathways in hepatocellular carcinoma (HCC), including gene variation, epigenetic inheritance, transcriptional regulation, post-transcriptional regulation, and post-translational modification. More studies have also found that the high expression of PD-L1 may be the main factor affecting the immunotherapy of liver cancer. However, what is the difference of PD-L1 expressed by different types of cells in the microenvironment of HCC, and which type of cells expressed PD-L1 determines the effect of tumor immunotherapy remains unclear. Therefore, clarifying the regulatory mechanism of PD-L1 in liver cancer can provide more basis for liver cancer immunotherapy and combined immune treatment strategy. In addition to its well-known role in immune regulation, PD-L1 also plays a role in regulating cancer cell proliferation and promoting drug resistance of tumor cells, which will be reviewed in this paper. In addition, we also summarized the natural products and drugs that regulated the expression of PD-L1 in HCC.

## 1 Introduction

Liver cancer is the third most common cause of cancer death worldwide (1). Hepatocellular carcinoma (HCC) accounts for 75% to 85% of primary liver cancers (2). Risk factors for liver cancer include hepatitis B virus (HBV), hepatitis C virus (HCV) infection, non-alcoholic fatty liver disease, alcohol consumption, type 2 diabetes, and aflatoxin (3). Early liver cancer is mainly treated by resection, local intervention or liver transplantation. However, most patients have advanced liver cancer, and even after treatment, patients will relapse or metastasize within 5 years, so systemic therapy is still the main means of liver cancer treatment (4). In recent years, targeted therapy based on anti-angiogenic drugs has been the first-line drug in the treatment of advanced liver cancer (5). However, clinical studies have shown that sorafenib can only extend the survival of advanced HCC by 3 months, and there are adverse events such as tolerance (6).

Recently, with the continuous research on the tumor immune microenvironment and the interaction between immune cells and tumor cells. The application of immune checkpoint inhibitors (ICIs) in immune and tumor cells is a major breakthrough in the treatment of many solid tumors (7). The study found that drugs targeting programmed cell death protein 1 (PD-1)/programmed cell death 1 ligand 1 (PD-L1) had significant anti-HCC effects (8). PD-1 is expressed on a variety of immune cells. By binding to ligand PD-L1 or programmed cell death 1 ligand 2 (PD-L2), PD-1 blocks the stimulation signal of T cell receptor (TCR), reduces the activity of T cells during immune response, and prevents autoimmune damage (9). In HCC, PD-L1 is mainly expressed on tumor cells, Kupffer cells, and hepatocytes (10). During immune activation, tumor antigens on cancer cells are presented to T cells by antigen-presenting cells (APC) and are recognized by binding to TCR. Activated T cells will release perforin, granzyme, interferon, and other cytokines to attack these cancer cells. And tumor cells escape T cell attack by expressing low levels of co-stimulatory immune checkpoint molecules, increasing inhibitory immune checkpoint molecules, such as PD-L1. Moreover, the increased expression of PD-L1 on tumor cells inhibits the anti-tumor effect and leads to immune tolerance of HCC (11). The expression of PD-L1 is closely related to the stage and poor prognosis of HCC (12). Therefore, it is important to clarify the regulatory mechanism of PD-L1 for HCC immunotherapy. We will review the expression and regulation of PD-L1 in different cells in the tumor microenvironment.

## 2 Expression of PD-L1 on host immune cells and tumor cells

Clinical patients with high expression of PD-L1 in liver tumor tissues have inconsistent responses to PD-1 inhibitors, which leads us to think about the expression of PD-L1 in tumor tissues. Through the literature research in the past decade, we found that there are new changes in the research of PD-L1. The attention of PD-L1 expression in tumor cells has gradually shifted to that of immune cells. In addition, we also found that immune cells and tumor cells are related, that is, changes in immune cell signaling regulatory factors can affect the expression of PD-L1 in tumor cells. Only by understanding the expression of PD-L1 in different types of cells and the relationship between them can immunotherapy for liver cancer be further advanced. We will summarize each of them.

### 2.1 Macrophages

**Figure 1:**
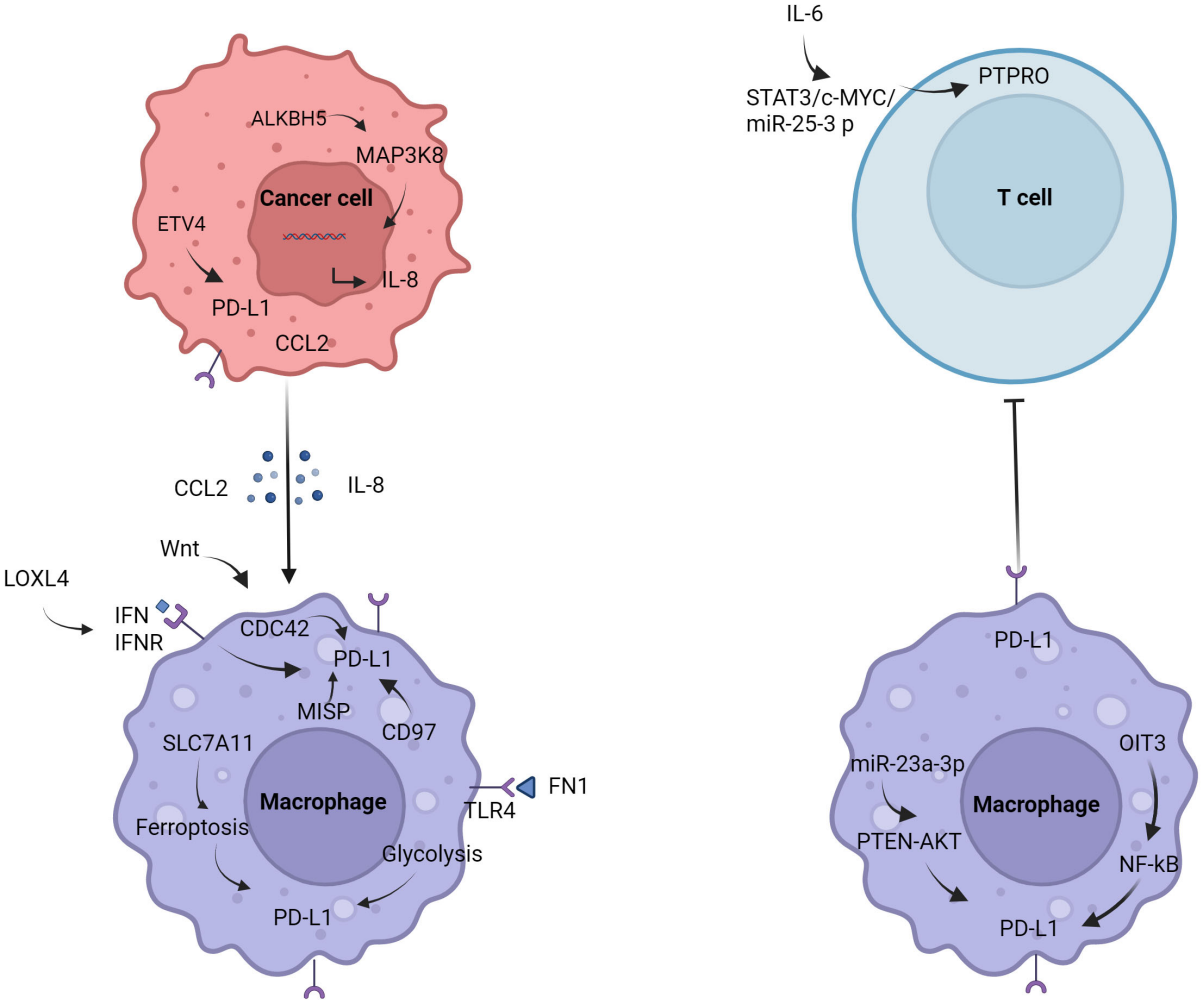
Regulation of PD-L1 in macrophages. Tumor cells induce the polarization of macrophages and promote the expression of PD-L1 by secreting IL-8 and CCL2. The expression of PD-L1 in macrophages is also affected by the glycolysis of FN1, IFN, PTEN, NF-κB, etc.

Studies have shown that macrophages in the tumor microenvironment can promote the growth of HCC (13). Macrophage surface expression of PD-L1 promoted the formation of immunosuppressive microenvironment (14). Regulation of PD-L1 in macrophages (**Figure 1**). It was found that AlkB homolog 5 (ALKBH5) promoted the recruitment of PD-L1^+^ macrophages mediated by interleukin-8 (IL-8) through mitogen-activated protein kinase kinase kinase 8 (MAP3K8), promoting HCC cell proliferation (15). Fibronectin 1 (FN1) promoted glycolytic activation of macrophages by triggering toll-like receptor 4 (TLR4), induced macrophages to express PD-L1 (16). Ferroptosis of macrophages mediated by solute carrier family 7a member 11 (SLC7A11) significantly increased the expression of PD-L1 in macrophages and improve the anti-tumor effect of anti-PD-L1 therapy (17). Low doses of interferon-α (IFN-α) also inhibited the growth of liver cancer in mice, possibly by polarizing CD169^+^ macrophage populations and enhancing CD8^+^ T cell activity. It was worth noting that IFN-α also induced a large amount of PD-L1 expression in macrophages *in vivo*, blocking PD-L1 further improved the anti-tumor effect of IFN-α (18). In addition, cell division cycle 42 (CDC42) was positively correlated with M2 macrophage markers and immune checkpoints, and the expression of CDC42 was most correlated with Wnt signaling pathway (19). The study also found that CD97 was positively correlated with M2 macrophages and tumor-associated macrophage markers, and positively correlated with PD-L1 (20). Lysyl oxidase-like 4 (LOXL4) was an amine oxidase, which was highly expressed in HCC tissues. LOXL4 promoted macrophage infiltration into the liver, accelerated tumor growth, and was further eliminated by adoptive T cell metastasis or PD-L1 neutralization. The immunosuppressive function of LOXL4 on macrophages was mainly dependent on IFN-mediated signal transduction and transcription-dependent activator of PD-L1 activation. Hydrogen peroxide scavenger or copper chelate macrophages eliminated PD-L1 presentation of IFN-mediated LOXL4 (21). Oncoprotein-induced transcript 3 (OIT3) mediated the polarization of macrophages and promoted the progression of HCC (22). OIT3 increased the expression of PD-L1 in TAMs by activating the nuclear factor kB (NF-κB) signaling pathway, blocked the immunosuppressive activity of NF-κB reversal TAMs, and inhibited the tumorigenesis of HCC (23). It was found that the expression of protein tyrosine phosphatase, receptor type O (PTPRO) was significantly decreased, which was related to the increase of PD-L1 expression in peripheral blood mononuclear cells and TAMs of HCC. Serum interleukin 6 (IL-6) decreased the expression of PTPRO by activating signal transducer and activator of transcription 3 (STAT3)/c-MYC/miR-25-3p axis, leading to PD-L1-induced immunosuppression to promote tumor growth (24). Endoplasmic reticulum (ER) stress occurred in HCC cells, released exosome miR-23a-3p, and upregulated the expression of PD-L1 in macrophages via miR-23a-PTEN-AKT pathway, and inhibited T cell function (25).

With the research on tumor immunotherapy in recent years, macrophages have gradually become the focus of research. In macrophages expressing LysM (lysozyme M), PD-L1 gene deletion eliminate the efficacy of anti-PD-L1 antibodies in MC38 colon cancer models (26). Macrophages in liver cancer may also have similar characteristics. How to regulate the expression of PD-L1, and then affect the polarization of macrophages, from promoting tumor progression to inhibiting tumor? Regulation of metabolic reprogramming of macrophages may be the future trend. These studies provided new insights into the mechanisms of how tumor cells escape from anti-tumor immunity.

### 2.2 Myeloid-derived suppressor cells

Myeloid-derived suppressor cells (MDSCs) may play an important role in immune regulation (27), but the immunosuppressive function of MDSCs in HCC patients has not been clarified. HCC cell lines with high expression of colony stimulating factor 1 (M-CSF) and vascular endothelial growth factor A (VEGFA) could significantly induce the expression of PD-L1 in MDSCs (28). It was found that MDSCs contributed to the formation of tumor immunosuppressive microenvironment. Tumor infiltrating CD11b^+^ CD33^+^ HLA-DR^-^MDSCs in HCC patients effectively inhibited the proliferation of CD8^+^ T cells. Studies have shown that cyclin dependent kinase 20 (CCRK) leads to MDSCs accumulation by activating the enhancer of zeste homolog 2 (EZH2)/NF-κB/IL-6 cascade (29). Notably, neoplastic CCRK depletion upregulated PD-L1 expression and increased intracellular CD8^+^ T cells, enhancing the effect of anti-PD-L1 in the treatment of liver cancer. Studies have found that SLC7A11 is significantly correlated with PD-L1 expression and adverse survival time (30). IL-1β-induced SLC7A11 over-expression promoted the infiltration of TAMs and MDSCs by up-regulating PD-L1 and colony stimulating factor-1 (CSF1) through the α-ketoglutarate (αKG)/hypoxia inducible factor-1α (HIF1α) axis (31).

### 2.3 Monocyte

Autocrine TNF-α and interleukin 10 (IL-10) released by activated monocytes stimulated the expression of PD-L1 in monocytes (32). PD-L1^+^ monocytes effectively inhibited tumor-specific T cell immunity and contributed to tumor growth in humans. Therefore, the expression of PD-L1 on activated monocytes/macrophages might represent a novel mechanism that links pro-inflammatory responses to immune tolerance in the tumor environment (32). IL-10 secreted by tumor monocytes was involved in the expression of PD-L1 on Treg cells through the JNK-STAT3 pathway (33). 6-phosphofructo-2-kinase/fructose-2,6-bisphosphatase 3 (PFKFB3) mediated the expression of PD-L1 by activating NF-κB signaling in the tumor microenvironment (34).

As one of the most abundant immune cells, neutrophils in the tumor microenvironment are involved in tumor progression, including promoting tumor invasion and metastasis, inhibiting adaptive immunity, and suppressing the anti-tumor response of T cells (35). Studies have shown that PD-L1^+^ neutrophils in HCC patients effectively inhibit the proliferation and activation of T cells, and blocking PD-L1 partially reverse this effect (36).

### 2.4 Regulation of tumor PD-L1 by immune cells

**Figure 2:**
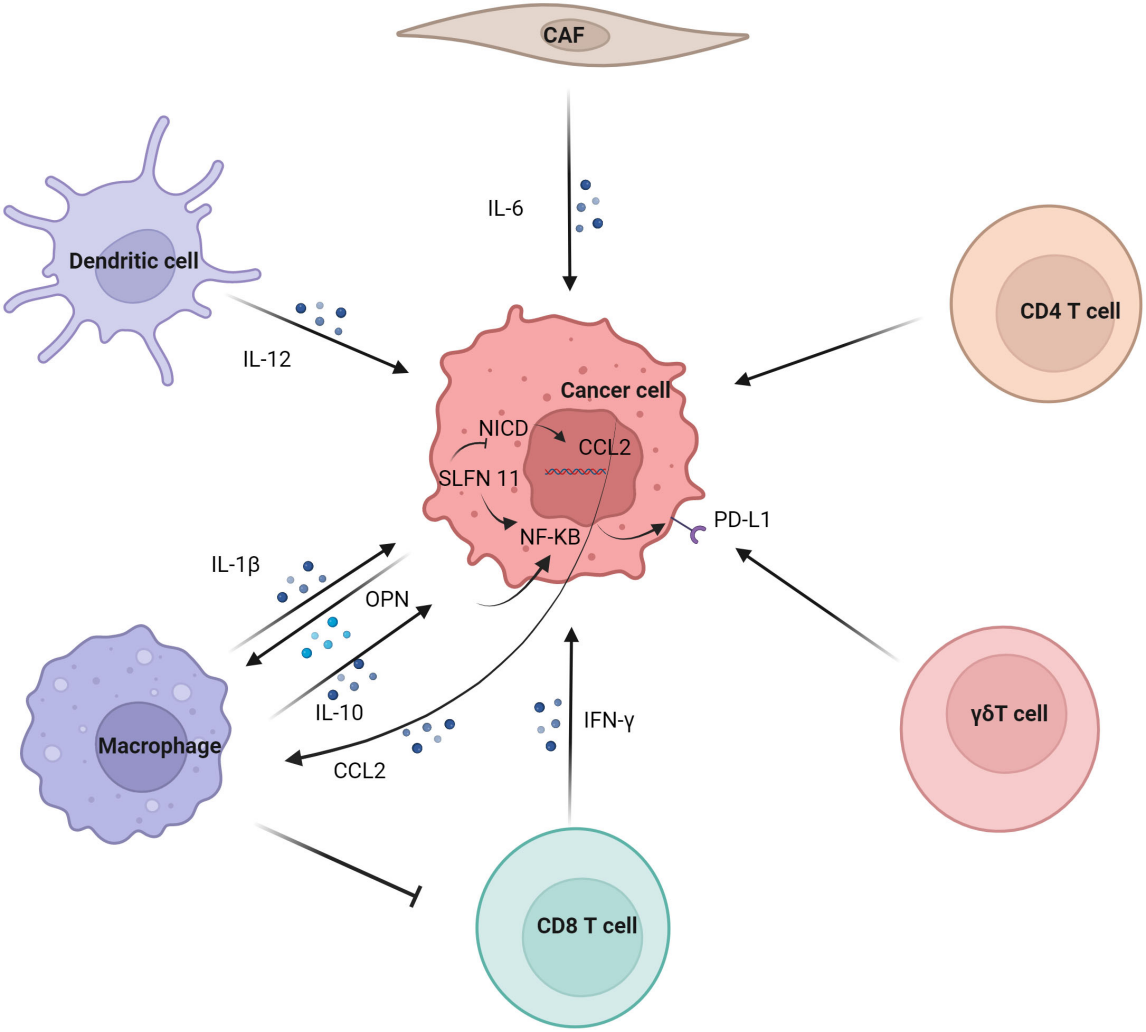
Regulation of tumor PD-L1 by immune cells. Dendritic cell, macrophage, CD8 T cell, γδT cell, CD4 T cell and CAF regulate the expression of PD-L1 on tumor cells. Macrophages have significant effect on the expression of PD-L1 in tumor cells, mainly involving IL-1β, IL10 and other cytokines on the NF-κB pathway in tumor cells.

Regulation of tumor PD-L1 by immune cells (**Figure 2**). In the tumor immune microenvironment, the carcinogenic activity of endogenous osteopontin (OPN) promoted chemotactic migration and substitution activation of macrophages. It also promoted PD-L1 expression in tumor cells by activating CSF1/CSF1R pathway in macrophages (37). Blocking CSF1/CSF1R prevents TAMs transport, enhancing the efficacy of ICIs in the treatment of HCC. Schlafen (SLFN) protein played an important role in cell proliferation and immune cell development (38). Studies have shown that macrophages induced by SLFN11 deficiency up-regulate the expression of PD-L1 in HCC cells through the NF-κB/P65 pathway. Blocking the CCL2 pathway enhanced the anti-PD-L1 efficacy of SLFN11 with low expression of HCC (39). Over-expression of e-twenty-six-specific sequence variant 4 (ETV4) in HCC cells activated the expression of PD-L1 and chemokine (C-C motif) ligand 2 (CCL2). The infiltration of tumor-associated macrophages (TAMs) and MDSCs was increased, and the accumulation of CD8^+^T cells was inhibited (40).

Although M1 macrophages are generally considered to have anti-tumor effects, some studies have reported their tumor-promoting effects (41). Studies have shown that the infiltration of CD68^+^ HLA-DR^+^ M1-like macrophages is related to the expression level of PD-L1 in HCC cells. The expression of tumor cell transcription factors NF-κB p65 and interferon regulatory factor-1 (IRF-1) induced by interleukin-1β (IL-1β) secreted by M1 macrophages promoted the expression of PD-L1 (42). M2 macrophage-derived extracellular vesicles (M2-EVs) up-regulated the expression of PD-L1 through the MISP/IQGAP1/STAT3 pathway, inhibited the killing ability of CD8^+^T cells and promoted immune escape of HCC cells (43).

The increased level of PD-L1 might represent the adaptive immune resistance mechanism generated by tumor cells in response to endogenous anti-tumor activity. PD-L1 up-regulation was mainly induced by pre-existing activated CD8^+^ T cells in HCC environment (44). It has also been suggested that PD-L1 down-regulates genes related to T cell activation in TME. Co-culture of PD-L1-expressing mouse liver cancer cell line BNL-MEA with CD8^+^ T cells reduced the proliferation of T cell and the expression of interferon γ (IFN-γ) and TNF-α (45). Although PD-L1-expressing tumors showed a better response to anti-PD-1 therapy, CD8^+^ T cells exhaustion inhibited the anti-PD-1 anti-tumor effect. Studies have shown an increase in circulating PD-1^+^CD8^+^ T cells in HCC. In addition, tumor-infiltrating CD8^+^T cells showed a sharp increase in PD-1 expression, which was associated with poor disease progression and postoperative recurrence. CD8^+^ T cells induced the expression of PD-L1 on liver cancer cells in an IFN-γ-dependent manner, promoting the apoptosis of CD8^+^ T cells (46). γδT cells infiltrate in liver cancer and have a strong killing ability (47). It was also found that co-incubation of γδT cells increased the expression of PD-L1 in HCC cell lines (48).

Studies have shown that IL-6 is mainly secreted by cancer-associated fibroblasts (CAF). Moreover, CAF with high IL-6 expression induced immunosuppression by recruiting immunosuppressive cells, such as myeloid suppressor cells. In addition, CAF with high expression of IL-6 also disrupted the function of tumor-infiltrating T cells by up-regulating the expression of PD-L1 (49).

Studies have shown that PD-L1 expression is positively correlated with FoxP3^+^ Treg cell infiltration, and patients with high PD-L1 expression have poor prognosis (50). IL-12 was a cytokine naturally secreted by activated dendritic cells and mononuclear/macrophages (51). Studies have found that long-term induction of liver IL-12 expression inhibits the growth of liver cancer. In addition, the tumors of non-responsive mice expressed more FoxP3^+^ Treg cells and higher inhibitory immune checkpoint molecules, such as PD-1, PD-L1, vascular endothelial-derived growth factor (VEGF), cytotoxic T-lymphocyte associated protein 4 (CTLA-4), indoleamine 2,3-dioxygenase (IDO) and IL-10 (52).

In summary, what should we focus on? Studies have also demonstrated that T cell regeneration in the tumor microenvironment is insufficient to mediate the preclinical efficacy of anti-PD-L1. It has also been found in other studies that the interaction between PD-1 and PD-L1 in tumor-draining lymph nodes can predict the clinical efficacy of ICIs in patients with metastatic melanoma but not in primary tumor tissue. This also suggests that interactions between T cells and antigen-presenting cells in tumor draining lymph nodes may be critical for the efficacy of anti-PD-L1 (53). The blocking of PD-1/PD-L1 in local drainage lymph nodes may be an important direction for our future clinical and basic research. It also triggered our thinking in the treatment of liver cancer, and we may also pay attention to the relationship between PD-1 and PD-L1 in the subsequent liver cancer research. Therefore, we should first review the PD-L1 expression of immune cells and tumor cells in the local microenvironment of liver cancer tumors. Through the above review, we have a clearer understanding of the regulatory mechanism of PD-L1 in different cells, which provides more application space for our follow-up exploration of existing and newly studied targeted drugs.

## 3 Expression of PD-L1 in tumor cells

Most of the observed expression levels of PD-L1 on tumor cells only consider the expression levels of tumor cells in a certain time and space, and these studies are far from reflecting the true expression status of PD-L1 in tumor cells. Generally speaking, the expression of PD-L1 in tumor cells can be caused by the increase of PD-L1 caused by changes in tumor cells’ own signaling pathways (54), or by changes in the external environment, including the influence of T cells (55), macrophages, dendritic cells and tumor-related fibroblasts on tumor PD-L1 expression. And these two different causes of tumor cells PD-L1 elevation, treatment methods are completely different. The high expression of PD-L1 in tumor cells, which we are concerned about, can be either a “cause” for promoting disease progression or a “result” of immunotherapy response. Next, we will focus on the impact of changes in tumor cells themselves on PD-L1.

### 3.1 Genomic variation of PD-L1

The frequency and prognostic significance of PD-Ls gene alterations in liver cancer remain unknown. The clinical relevance and prognostic value of 9p24.1 gene alteration in an independent cohort of HCC patients were studied by tissue microarray analysis, and the results showed that the genetic alteration of 9p24.1 significantly promoted the upregulation of PD-L1 and PD-L2 (56). Nucleostemin (NS) promoted liver regeneration through damage repair mechanisms and protects human HCC cells from replication and drug-induced DNA damage. NS consumption in liver cancer cells increased physical DNA damage and the amount of cytoplasmic double-stranded DNA, leading to increased cytokine and PD-L1 reactivity (57).

### 3.2 Epigenetic regulation of PD-L1

Epigenetic regulation of PD-L1 expression (**Table 1**).

**Table 1:** Epigenetic regulation of PD-L1 expression.

Key molecular	Regulation mechanism	PD-L1 changes	References
HDAC	HDAC up-regulates PD-L1	Up-regulation	(58)
EZH2	EZH2 can inhibit PD-L1 expression by upregulating lysine trimethylation level at N-terminal 27 of histone H3 and IRF1 on CD274 promoter	Down-regulation	(59)
PRMT1	Loss of PRMT1 reduces PD-L1 expression in tumors	Up-regulation	(60)
DNMT1	DNMT1 is positively correlated with PD-L1 over-expression	Up-regulation	(61)
PD-L1L2-SE	Activation of PD-L1L2-SE was required for the expression of PD-L1 and PD-L2 in tumor cells	Up-regulation	(62)
LRPPRC	LRPPRC might partially up-regulate the post-transcriptional expression of PD-L1 in an m6A-dependent manner	Up-regulation	(63)
ALKBH5	ALKBH5 inhibited the expansion and cytotoxicity of T cells by sustaining tumor cell PD-L1 expression	Up-regulation	(64)

#### 3.2.1 Histone acetylation

Many studies have shown that histone deacetylation regulates the expression of immune checkpoints and plays an important role in cancer progression (65). Gasdermin D (GSDMD) inhibited cGAS activation by promoting autophagy through the output of potassium (K^+^). The expression of PD-L1 was promoted by histone deacetylase/signal transducer and activator of transcription 1 (STAT1), which induced the counter-activation of PD-L1 by input calcium (Ca^2+^) (66). Studies have shown that histone deacetylase (HDAC) makes cancer cells sensitive to ICIs therapy by up-regulating the expression of CTLA-4, PD-1, PD-L1, and PD-L2 on tumor cells and tumor infiltrating lymphocytes (TILs) (58). In addition, the epigenetic regulation of immune checkpoints molecules used to improve the tumor microenvironment also expands the understanding of potential therapeutic targets for improving the tumor microenvironment and restoring immune recognition and immunogenicity (67). Recently, *in vitro* and *in vivo* results have shown that epigenetic modifiers play an important role in triggering and enhancing the host immune system in the treatment of cancer (68). Two important epigenetic mechanisms in cancer included hypermethylation mediated by DNMT and histone deacetylation mediated by HDAC. Some epigenetic regulators played a negative role in the immune response, inducing immune escape in cancer cells (69). Two important epigenetic drugs, histone deacetylase inhibitor (HDACI) and DNA methyltransferase inhibitors (DNMTIs), up-regulated the expression of immune checkpoints molecules in immune cells or cancer cells (68). This provides a new mechanism for ICIs to treat cancer.

#### 3.2.2 Histone methylation

EZH2 inhibited the expression of PD-L1 in HCC cell lines by up-regulating the promoter trimethylation on histone 3 lysine 27 (H3K27me3) (59). EZH2 might be a potential therapeutic target for the combination therapy of immune therapy for HCC. In addition, protein arginine methyltransferase 1 (PRMT1) specifically methylated the 3-site arginine of histone H4 *in vitro* and *in vivo*. Deletion of PRMT1 in mice reduced the expression of PD-L1 and PD-L2 in tumors and reduced the therapeutic effect of anti-PD-1 in HCC mice (60).

#### 3.2.3 DNA methylation

Studies have shown that features of HCC and T cell DNA methylation are widespread in peripheral blood mononuclear cells (PBMC) and are highly enriched in genes associated with immune function. For example, PD-1 (70). Studies have shown that the significant up-regulation of DNMT1 is positively correlated with PD-L1 over-expression in sorafenib resistant HCC cells (61).

#### 3.2.4 Super-enhancer

Super-enhancers are defined DNA regulatory elements that can be distinguished from enhancers through the size of DNA elements and epigenetic modifications such as H3K4me1, H3K4me3, and H3K27Ac (71). Super-enhancers are extremely important to maintain cell identity through inducing the expression of pivotal lineage-specific genes. By hijacking this mechanism, tumor cells often assemble new super-enhancers to trigger oncogenes such as MYC(72, 73). The SPACE prediction model also successfully predicted the super enhancer of PD-L1 (74). Activation of PD-L1L2-SE was required for the expression of PD-L1 and PD-L2 in tumor cells. Deletion of the PD-L1L2-SE gene caused tumor cells to lose immune escape and made them sensitive to T cell killing. PD-L1 and PD-L2 induced by PD-L1L2-SE were not associated with IFN-γ. Therefore, epigenetic activation of this region (PD-L1L2-SE) was associated with PD-L1 and PD-L2 (62). Studies have shown that these enhancers can predict prognosis better than nearby genes (75).

#### 3.2.5 N6-methyladenosine

N6-methyladenosine (m6A) is a novel epigenetic modification and an important regulator of HCC progression (15). Leucine rich pentatricopeptide repeat containing (LRPPRC)-mediated M6A modification had important effect on PD-L1 mRNA and immune escape in HCC (63). LRPPRC might partially up-regulate the post-transcriptional expression of PD-L1 in an m6A-dependent manner, enhancing the stability of PD-L1 mRNA (63). In addition, tumor-intrinsic ALKBH5 inhibited the expansion and cytotoxicity of T cells by sustaining tumor cell PD-L1 expression (64).

### 3.3 Transcriptional regulation of PD-L1

**Figure 3:**
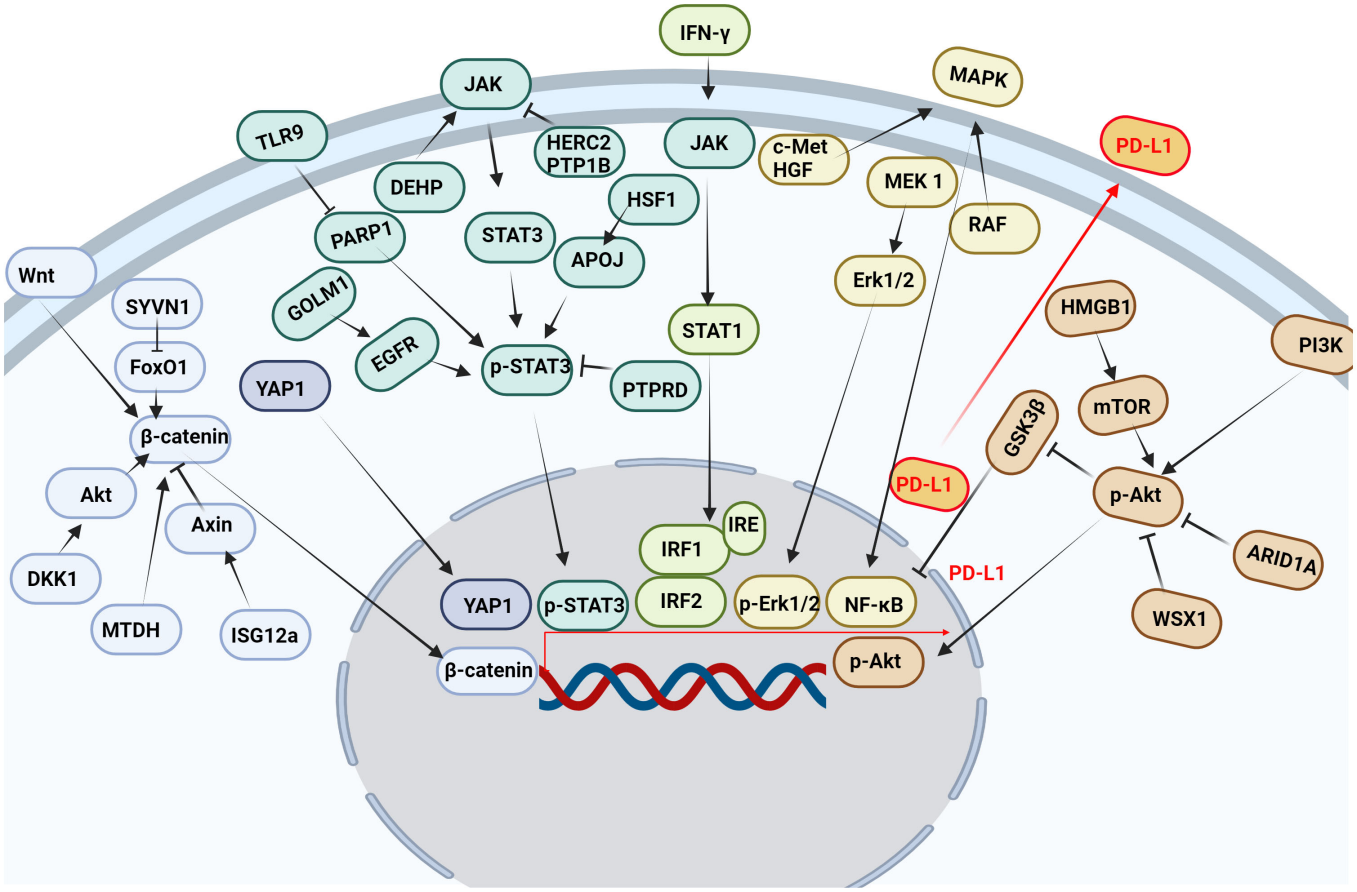
Multiple pathways and targets regulate the transcription of PD-L1. Wnt, Hippo, JAK, IFN-γ, MAPK, PI3K and other signaling pathways affect the transcription of PD-L1 in tumor cells.

Multiple pathways and targets regulate the transcription of PD-L1 (**Figure 3**).

#### 3.3.1 JAK/STAT signaling pathway

The JAK/STAT signaling pathway is abnormally activated in HCC, and its downstream target genes control dysfunctions of tumor growth and angiogenesis, invasion, and metastasis (76). It was found that STAT3 bound to PD-L1 promoter and transcriptionally to regulate PD-L1 expression (77). Decreased STAT3 activity led to decreased IFN-γ-induced PD-L1 expression and restored T cell sensitivity (78). In addition, the phosphorylation of the upstream molecular pathway of STAT3 also affected the expression of PD-L1. Protein tyrosine phosphatase receptor delta (PTPRD) inhibited the expression of PD-L1 by inhibiting the phosphorylation of STAT3 (79). Golgi membrane protein 1 (GOLM1), as an oncogene, promoted the growth and metastasis of liver cancer by selectively binding with epidermal growth factor receptor (EGFR) (80). In addition, GOLM1 promoted the phosphorylation of STAT3 by enhancing the level of EGFR, up-regulating the transcriptional expression of PD-L1 (80). Toll-like receptors 9 (TLR9) negatively regulated the expression of PARP1 mediated the decrease of STAT3 Poly (ADP-ribosyl) ation (PARylation) and the increase of STAT3 Tyr705 phosphorylation, and promoted the transcription of PD-L1 (81). Studies have shown that HECT domain and RCC1-like domain 2 (HERC2) enhance cancer stemness and PD-L1-mediated immune escape of HCC cells, which is associated with activation of the STAT3 pathway during the inflammation-cancer transformation. Coupling of HERC2 with endoplasmic reticulum (ER)-resident protein tyrosine phosphatase 1B (PTP1B) restricted PTP1B transport from the ER to the ER-plasma membrane junction, improving inhibitory effect of PTP1B on phosphorylation of Janus kinase 2 (JAK2). In addition, HERC2-knocked out hepatocytes limited hepatic PD-L1 expression and improved HCC progression (82). Heat shock factor 1 (HSF1) up-regulated PD-L1 expression by inducing APOJ expression and activating STAT3 signaling pathway (83). Di (2-ethylhexyl) phthalate (DEHP) might promote the expression of PD-L1 by up-regulating JAK2/STAT3 levels, inhibiting anti-tumor immunity (84).

#### 3.3.2 IFN-γ signaling pathway

PD-L1 expression was primarily induced by IFN-γ released from tumor-infiltrating T cells in HCC (85). IFN-γ induced PD-L1 expression by up-regulating IRF-1 expression in mouse and human HCC cells (86) (87). It has also been found that IFN-γ induces PD-L1 expression through the JAK/STAT1/IRF1 pathway in HCC cell lines (88). Both the transcription factors IRF-1 and IRF-2 signaling pathways regulated PD-L1 in HCC cells. IRF-1 antagonized IRF-2 binding to IRE promoter in PD-L1, providing new insights into the regulation of PD-L1/PD-1 pathway during ICIs therapy of HCC. In addition, over-expression of IRF-2 inhibited IFN-γ-induced PD-L1 promoter activity and protein levels (87). Studies have shown that TNF-α enhances IFN-γ signaling by up-regulating the expression of IFN-γ receptor. In addition, the expression of PD-L1 induced by TNF-α and IFN-γ promoted the growth of liver cancer (88). IFN-γ and IL-1β have a synergistic effect on PD-L1 expression (89).

#### 3.3.3 Wnt/β-catenin signaling pathway

β-catenin was highly expressed in a variety of tumors and played an important role in tumor growth, metastasis and recurrence, especially in HCC patients. And the nuclear accumulation of β-catenin in cancer cells often predicted a poor prognosis (90). Studies have shown that interferon stimulated gene 12a (ISG12a) promotes β-catenin proteasome degradation by inhibiting ubiquitination degradation of Axin, thereby inhibiting Wnt/β-catenin signaling (91). β-catenin was considered to be a transcription factor of PD-L1.ISG12a inhibited the expression of PD-L1 by inhibiting Wnt/β-catenin signaling, rendering cancer cells sensitive to NK cell-mediated killing (92). Studies have shown that dickkopf-1 (DKK1) is positively correlated with PD-L1 and negatively correlated with CD8^+^ T cell infiltration in human HCC. Overexpression of DKK1 promoted PD-L1 expression by activating Akt/β-catenin signaling pathway (93). Metadherin (MTDH) increased PD-L1 expression and up-regulated PD-L1 transcriptional activity through β-catenin/LEF-1 signaling pathway. More importantly, MTDH ASO improved anti-PD-1 response in PD-1-treated malignancies and increased infiltration of cytotoxic T cells (94). PD-L1 up-regulated serum and glucocorticoid kinase 2 (SGK2), activated SGK2/β-catenin signaling pathway, and promoted the expansion of HCC cell epithelial-mesenchymal transition (EMT) and cancer stem cell (CSC) (95). Synoviolin (SYVN1) regulated FoxO1 ubiquitination and stimulated β-catenin nuclear translocation, promoting PD-L1-mediated liver cancer metastasis and immune escape (96).

#### 3.3.4 MAPK signaling pathway

Mitogen-activated protein kinase (MAPK) signaling pathway was associated with the expression of PD-L1 in liver cancer (97). Epidermal growth factor (EGF) or IFN-γ promoted the increase of PD-L1 in HCC cell lines. While EGFR and mitogen-activated protein kinase kinase 1 (MEK1) and mitogen-activated protein kinase kinase 2 (MEK2) were blocked, EGF and IFN-γ-induced up-regulation of PD-L1 was inhibited. In addition, IFN-γ increased the transcriptional activity of PD-L1, while MAPK signaling increased the stability of PD-L1 mRNA (97). Moreover, MET proto-oncogene, receptor tyrosine kinase (c-Met) was a receptor for hepatocyte growth factor/scatter factor (HGF/SF). HGF induced c-Met activation occurs during the activation of the PD-1/PD-L1 signaling pathway (98). As the upstream target molecule of PD-L1, c-Met regulated the transcription of PD-L1 through the MAPK/NF-кBp65 pathway, promoting the progression of HCC (99). In addition, it was found that trans-activation of RAF dimer and ERK signal promoted HCC cell survival and PD-L1 expression through MAPK/NF-κB pathway (100). Studies have shown that blocking IKK complex formation leads to reduced nuclear translocation of NF-κBp65 and PD-L1 expression (101). It was found that up-regulation of alpha fetoprotein (AFP) increased the expression of PD-L1 in HCC tissues by activating P65 protein (102).

#### 3.3.5 PI3K/AKT pathway

The PI3K/AKT signaling pathway participates in the growth and metastasis of HCC (103). Studies have shown that the RNA-RNA crosstalk network driven by high mobility group box-1 (HMGB1) promotes glutamine metabolism in HCC cells through a dual mechanism. Activation of mTORC2-AKT-C-MYC positive feedback loop up-regulated glutamine synthetase (GS) expression and induced inhibition of SIRT4 on glutamate dehydrogenase (GDH) by mTORC1 signaling pathway. At the same time, this crosstalk network may hinder the efficacy of immunotherapy through mTORC1-P70S6K-dependent PD-L1 production and PD-L1^+^ exosome activity (104). It was found that the absence of AT-rich interaction domain 1A (ARID1A) activated phosphatidylinositol-4,5-bisphosphate 3-kinase (PI3K)/AKT signaling was significantly correlated with the high expression of PD-L1 in HCC. Low expression of ARID1A and high expression of PD-L1 were independent prognostic factors for overall survival (OS) and relapse-free survival (RFS). Patients with ARID1A deletion and high expression of PD-L1 had the worst prognosis. HCC with low expression of ARID1A was significantly associated with high levels of tumor-associated CD68-positive macrophages (105). WSX1 was down-regulated in HCC cells, and WSX1 enhanced hepatic immune surveillance by blocking the PI3Kδ/AKT/GSK3β/PD-L1pathway (106).

#### 3.3.6 Hippo pathway

Hippo signaling pathway inactivation induces the activation of yes-associated transcriptional regulatory factor 1 (YAP1), which regulates gene transcription (107). Studies have found that YAP1 inhibitors reduce the expression of PD-L1 in tumor tissues, promote the infiltration of CD8 T cells and CD4 T cells into tumor tissue (108), which disrupt the immunosuppressive microenvironment of cancer and improve the efficacy of HCC treatment (109). Interestingly, our research group also found that PD-1/PD-L1 interaction up-regulates YAP1 expression in HepG2 cells through the MAPK/ERK pathway (110). M2-polarized macrophages stimulated by IgA complex activated YAP/TAZ mediated signaling pathway, inducing cell activation and PD-L1 up-regulation *in vitro* (111).

#### 3.3.7 EMT

EMT was a malignant tumor phenotype characterized by invasion and metastasis, and TNF-α-induced EMT led to up-regulation of immunomodulators, including PD-L1 and PD-L2. Conversely, inhibition of EMT decreased the expression of PD-L1 and PD-L2 (112). In addition, TGF-β and fibroblast growth factor 2(FGF-2) effectively induced EMT through SMAD family member 3(SMAD3), MEK/Erk and mTOR pathways in HCC-827 cell line. Reversal of EMT partially restored chemical sensitivity and inhibited PD-L1 expression (113). It was found that TGF-β1 in HCC promoted EMT and induced the expression of PD-L1. TGF-β-specific inhibitor SB431542 blocked TGF-β1-mediated EMT and inhibited the expression of PD-L1 in liver cancer cells. Furthermore, down-regulation of PD-L1 inhibited EMT (114). During TGF-β1-induced EMT, the immune checkpoint molecules PD-L1 and B7-H3 were up-regulated. And reversing EMT decreased the expression of PD-L1 and B7-H3 (115).

#### 3.3.8 AR

Studies have shown that androgen receptor (AR) negatively regulates the expression of PD-L1 by acting as a transcription suppressor of PD-L1. Thus, AR inhibited the expression of PD-L1, possibly contributing to sex differences in HCC (116, 117). In addition, it was found that hydroxysteroid 17-beta dehydrogenase 6 (HSD17B6) played an important role in the occurrence and development of HCC. HSD17B6 inhibited the expression of transforming growth factor beta 1 (TGF-β1) and PD-L1 by transforming DHT (118).

#### 3.3.9 Other specific transcriptional regulation

SRY-box transcription factor 2 (SOX2) was a transcription factor that controls the expression of many target genes by forming trimer complexes with octamer-binding transcription factor 4 (OCT4) on DNA (119). SOX2 regulated the expression of PD-L1 by directly binding SOX2 common binding sites on the PD-L1 promoter region and regulating the promoter activity of PD-L1 (120). Y-box binding protein 1 (YB-1) 1 promoted the expression of multiple resistance genes, thus enhancing the drug resistance of tumors (121). It was found that chemotherapy induced immunosuppressive microenvironment formation and tumor immune escape through YB-1-mediated increase in PD-L1 (122). Oxidative stress responsive kinase 1 (OXSR1) was closely related to malignant progression of malignant tumors (123). The high expression of OXSR1 was positively correlated with the infiltration level of tumor-infiltrating immune cells (TIICs) and the expression of PD-L1 in HCC (124). In liver cancer cells, MYC up-regulated the expression of PD-L1 in lymphoma. Knocking down the expression of MYC promoted the increase of PD-L1 expression level (125). Down-regulation of C-X-C chemokine receptor 2 (Cxcr2) reduced PD-L1 levels and thus promoted the transformation of macrophages to the M1 type, which was mediated by down-regulation of MYC (126). The activation of the inhibitor of differentiation or DNA binding 1 (ID1)/MYC signal promoted immune escape and tumor progression of drug-resistant HCC through PD-L1 up-regulation and CCL5-induced PMN-MDSC recruitment in HCC cells (127). Methyltransferase-like 5 (METTL5) expression was elevated in HCC tissues and cells and was associated with poor prognosis. Down-regulation of METTL5 inhibited the expression of PD-L1 and the malignant cell behavior of HCC by inhibiting the MYC pathway (128). Inhibition of MYC increased the expression of STAT1, leading to increased PD-L1 expression in HCC cells exposed to IFN-γ (125). Anti-silencing function 1b (ASF1B) was highly expressed in tumor tissues, which was correlated with poor OS and progression-free survival (PFS). And ASF1B was positively correlated with PD-L1 expression (129). CKLF-like MARVEL transmembrane domain–containing 4 (CMTM4) was the main regulator of PD-L1 in HCC. CMTM4 might stabilize PD-L1 and promote the escape of T-cell-mediated cytotoxicity (130). CKLF-like MARVEL transmembrane domain-containing protein 6 (CMTM6) maintained the expression of PD-L1 by controlling its systemic circulation (131). The high expression of CMTM6/PD-L1 was associated with poorer RFS and OS in HCC patients (132). Patients with CMTM6/PD-L1 co-expressed macrotrabecular-massive (MTM) HCC had a higher risk of disease progression and death (133). Increased expression of PD-L1 and AR-VRK2 induces immune escape, development and metastasis of liver cancer (134). The expression of human endogenous retrovirus-H long terminal repeat-associating protein 2 (HHLA2, also known as B7-H7) was negatively correlated with PD-L1. Patients with HHLA2 and PD-L1 co-expression had the shortest survival time (135). The expression of neurotrophic factor-3 (NTF3) was negatively correlated with PD-L1, T cell immunoreceptor with Ig and ITIM domains (TIGIT) and T cell immunoglobulin and mucin domain 3 (TIM-3) (136). MMP-12 might promote the development of HCC by up-regulating PD-L1 (137). Silencing RAB42 down-regulated PD-L1 expression and inhibited immune escape by inhibiting E2F signaling pathway in hepatoma cells (138). LW6 inhibited tumor angiogenesis, down-regulated the expression of PD-L1, and promoted the apoptosis of HCC cells by inhibiting HIF-1α (139).

### 3.4 Regulate the expression of PD-L1 after transcription

#### 3.4.1 Regulation of PD-L1 by microRNAs

Non-coding RNAs regulates the expression of PD-L1 (**Table 2**). MicroRNAs (miRNAs) were a class of endogenous non-coding RNAs that regulate cell cycle, proliferation and apoptosis, and their abnormal expression was associated with the occurrence of liver cancer (163). microRNA-1 (miR-1) was a tumor suppressor miRNA. MiR-1 directly regulated the expression of PD-L1, and the loss of miR-1 contributed to the upregulation of PD-L1 in sorafenib resistant liver cancer cells (140). Nuclear factor E2-related factor (Nrf-2) inhibited the expression of miR-1, and the regulatory axis of Nrf-2/miR-1/PD-L1 contributed to the maintenance and development of sorafenib resistance in HCC cells. In addition, it was found that miR-155-5p and miR-194-5p could up-regulate the expression of PD-L1 through X inactive specific transcript (XIST) (141). HOXA-AS3 increased PD-L1 expression. In addition, both inhibition of PD-L1 and overexpression of miR-455-5p reversed the effects of cell proliferation and invasion induced by HOXA-AS3 overexpression. (142). Olaparib enhanced the expression of PD-L1 in HCC cells by inhibiting miR-513. Inhibition of poly (ADP-ribose) polymerase (PARP) enhanced ICIs in HCC through the miR-513/PD-L1 pathway (143). Studies have shown that IRF-1 up-regulation induces HCC apoptosis by promoting miR-195 and inhibiting the expression of checkpoint kinase 1 (CHK1). IRF-1 expression or CHK1 inhibition also promoted PD-L1 expression by increasing STAT3 phosphorylation (144). MiR-329-3p inhibited the immunosuppression of tumor cells and enhanced the response of tumor cells to T-cell-induced cytotoxicity through reducing the expression of lysine-specific demethylase 1A (KDM1A, also known as LSD1).Thus, myocyte enhancer factor 2D (MEF2D) demethylation and PD-L1 expression activation were promoted (145). Studies have shown that down-regulation of myocardial infarction-associated transcript (MIAT) can enhance the cytotoxicity of T cells to HCC cells and increase the expression of miR-411-5p, STAT3 and PD-L1. Inhibition of miR-411-5p reversed the expression of STAT3 and PD-L1 in HCC cells inhibited by MIAT knockout (146). MiR-378a-3p mimics effectively reduced the expression of and inhibited the differentiation of Tregs in co-culture models. In addition, overexpression of miR-378a-3p inhibited cell proliferation and migration in HCC cells, while promoted apoptosis by inhibiting STAT3 signaling (147). β-Glucuronidase (GUSB) promoted the proliferation, invasion and migration of human HCC cells by promoting miR-513a-5p.It also down-regulated the expression of PD-L1, resulting in primary resistance to anti-PD-1 therapy (148). In HCC, p-P38 mitogen activated protein kinase (MAPK) increased activation and down-regulated miR-675-5p. Down-regulation of miR-675-5p might enhance the stability of PD-L1 mRNA through the 3’-untranslated region (3’-UTR) of PD-L1, resulting in the accumulation of PD-L1. Upregulation of HK2 enhanced aerobic glycolysis and mediated the decrease of HLA-ABC (149). These results suggested that miRNAs might play an important role in the immune microenvironment of HCC and had certain guiding significance for the clinical treatment of HCC.

**Table 2:** Non-coding RNAs regulation of PD-L1 expression.

Key molecular	Regulation mechanism	PD-L1 changes	References
MiR-1	Inhibits the expression of PD-L1	Down-regulation	(140)
MiR-155-5p and miR-194-5p	MiR-155-5p and miR-194-5p can up-regulate PD-L1 through XIST	Up-regulation	(141)
MiR-455-5p	Both PD-L1 inhibition and miR-455-5p overexpression reversed cell proliferation and invasion induced by HOXA-AS3 overexpression	Down-regulation	(142)
MiR-513	Olaparib enhances the expression of PD-L1 in HCC cells by inhibiting miR-513	Down-regulation	(143)
MiR-195	Promotes the expression of PD-L1	Up-regulation	(144)
MiR-329-3p	MiR-329-3p promotes MEF2D demethylation and activation of PD-L1 expression	Up-regulation	(145)
MiR-411-5p	Inhibition of miR-411-5p can reverse the expression of STAT3 and PD-L1 in HCC cells inhibited by MIAT knockout	Down-regulation	(146)
MiR-378a-3p	MiR-378a-3p mimics can effectively reduce the expression levels of PD-L1 mRNA and protein by regulating the expression levels of certain cytokines	Down-regulation	(147)
MiR-513a-5p	MiR-513a-5p promotes the proliferation, invasion and migration of human HCC cells, and down-regulated the expression of PD-L1	Down-regulation	(148)
MiR-675-5p	Downregulation of miR-675-5p enhanced the stability of PD-L1 mRNA through the 3’ -UTR of PD-L1, causing the accumulation of PD-L1	Down-regulation	(149)
PCED1B-AS1	Promotes the expression of PD-L1	Up-regulation	(150)
LncRNA MIAT	Promotes the expression of PD-L1	Up-regulation	(151)
Lnc-RAB11B	High expression of RAB11B may lead to downregulation of PD-L1	Down-regulation	(152)
LncRNA AC099850.3	Promotes the expression of PD-L1	Up-regulation	(153)
LINC00657	LINC00657 inhibits PD-L1 expression by decreasing miR-424	Down-regulation	(154)
LncRNA KCNQ1	Promotes the expression of PD-L1	Up-regulation	(155)
LINC00244	Inhibits the expression of PD-L1	Down-regulation	(156)
LINC00638	The LINC00638/miR-4732-3p/ULBP1 axis promotes immune escape of HCC through PD-L1	Up-regulation	(157)
Hsa-circ-0006852	Hsa-circ-0006852 promotes the increase of PD-L1 expression by activating the NF-κB signaling pathway	Up-regulation	(158)
Hsa-circ-0003288	Hsa-circ-0003288 acts as a miR-145 sponge and up-regulates PD-L1 expression through the PI3K/AKT signaling pathway	Up-regulation	(159)
Has-circ-0005239	Inhibits the expression of PD-L1	Down-regulation	(160)
CircWDR25	CircWDR25 promotes the expression of PD-L1 in HCC cells through circWDR25/miR-4474-3p/ALOX15 and EMT axis	Up-regulation	(161)
CircPRDM4	CircPRDM4 promotes PD-L1 expression by promoting HIF-1α recruitment to CD274 promoter	Up-regulation	(162)

#### 3.4.2 Regulation of PD-L1 by lncRNAs

Long noncoding RNAs (lncRNAs) are a class of non-coding RNAs that have limited protein-coding ability and are involved in the genesis and development of tumors (164). Many studies have demonstrated that lncRNA can regulate PD-L1 expression. In HCC, the expression of PCED1B antisense RNA1 (PCED1B-AS1) and hsa-miR-194-5p was up-regulated in lncRNA. In liver cancer, PCED1B-AS1 interacted with hsa-miR-194-5p, which inhibited the expression of PD-Ls, and enhanced the expression of PD-Ls (150). Cancer susceptibility 11 (CASC11) recruited eukaryotic translation initiation factor 4A3 (EIF4A3) and enhanced the stability of E2F1 mRNA. It further affected the NF-κB signal and promoted the activation of PI3K/AKT/mTOR pathway, regulating the expression of PD-L1 (165). Studies have shown that the expression of LncRNA MIAT in liver cancer is positively correlated with the expression of inhibitory immune checkpoint molecules such as PD-1, PD-L1 and CTLA4 (151). Low expression of lnc-RAB11B-AS1 was associated with shorter OS and DFS in HCC patients. The high expression of RAB11B reduced PD-L1 expression, thereby inhibiting the progression of HCC (152). LncRNA AC099850.3 was up-regulated in HCC tissues, and its high expression was associated with poor prognosis in HCC patients. LncRNA AC099850.3 significantly improved the proliferation and invasion ability of HCC cells through the PRR11/PI3K/AKT pathway. In addition, lncRNA AC099850.3 affected the abundance of various immune cells in the tumor microenvironment, especially M2 macrophage infiltration, and was positively correlated with PD-L1 (166). LINC00657 was highly expressed in HCC and was associated with poor prognosis. LINC00657 regulated the expression of PD-L1 by decreasing miR-424. The 3’UTR of PD-L1 was highly conserved with that of miR-424, and miR-424 significantly inhibited the mRNA and protein levels of PD-L1 (154). LncRNA KCNQ1 overlapping transcript 1 (lncRNA KCNQ1OT1) was closely related to drug resistance in cancer. KCNQ1OT1 acted as a competitive endogenous RNA of miR506 and increased PD-L1 expression in sorafenib resistant HCC cells (155). LINC00244 inhibited HCC proliferation, invasion and metastasis by down-regulating the expression of PD-L1. In addition, low expression of LINC00244 activated the EMT pathway, promoting rapid growth and metastasis of HCC cells (156). Lipopolysaccharide (LPS) induced the expression of PD-1 and PD-L1 in mouse tumor and induced the expression of PD-L1 in HCC cells. LPS played a key role in immune escape of HCC through the METTL14/MIR155HG/PD-L1 axis (167). Studies have shown that patients with high ULBP1 and PD-L1 have the worst prognosis. The LINC00638/miR-4732-3p/ULBP1 axis associated with tumor mutation burden promoted immune escape in HCC via PD-L1 (157).

#### 3.4.3 Regulation of PD-L1 by circRNAs

Circular RNAs (circRNAs) were non-coding RNAs with a closed-loop structure that regulated biological processes by acting as sponges for miRNAs or binding to proteins, and many circRNAs were involved in cell proliferation and invasion of HCC (168). In recent years, the effect of circRNAs mediated PD-L1 expression on the immune status of liver cancer has attracted much attention. Has-circ-0006852 (circCORO1C) promoted the development of HCC by activating the NF-κB signaling pathway, increasing the phosphorylation of P65, the expression of c-Myc, COX-2 and PD-L1 (158). Has-circ-0003288 acted as a miR-145 sponge and up-regulated PD-L1 expression through the PI3K/AKT signaling pathway, promoting EMT and HCC invasion (159). Targeting has-circ-0003288 might provide a therapeutic strategy for the treatment of HCC. Has-circ-0005239 promoted migration, invasion, and angiogenesis by controlling PD-L1 expression in HCC. These results revealed that has-circ-0005239 might be a potential therapeutic target for patients with advanced HCC (160). Exogenous and hepatic stellate cell (HSC) exosome derived circWDR25 promoted the proliferation and invasion of HCC cells through the circWDR25/miR-4474-3p/ALOX15 and EMT axes. It also promoted the expression of CTLA-4 and PD-L1 in HCC cells (161). CircPRDM4 was a circRNA, which was associated with hypoxia in HCC. CircPRDM4 promoted PD-L1 activation by promoting HIF-1α recruitment to the PD-L1 promoter and consolidating their interaction under hypoxic conditions. Thus, CD8^+^ T cell infiltration was inhibited and immune escape of HCC cells was increased (162).

These studies suggested that miRNAs, lncRNAs and circRNAs directly displayed epigenetic functions by recruiting specific protein complexes into genomic DNA, especially certain promoters that regulated the expression of corresponding genes. Studies have also shown that miRNAs, lncRNAs and circRNAs play an important role in regulating the expression of immune checkpoint molecules in various tumors (169, 170). Whether the association between miRNA expression and immune checkpoint levels in tumors can be translated into predictive markers for checkpoint inhibitor therapy in liver cancer needs further investigation. The interaction between the three RNAs was revealed in the “lncRNA-miRNA-mRNA” competitive endogenous RNA network. Some miRNAs and lncRNAs participated in the “cancer immune cycle” regulated by immune checkpoints molecules and had the potential to be the subject of future research in liver cancer.

### 3.5 Regulation of PD-L1 by post-translational modification

#### 3.5.1 Phosphorylation regulation

EGF treatment enhanced H3-Thr11 phosphorylation at the PD-L1 promoter and promoted the expression of PD-L1 in HCC cells. Inhibition of EGFR reversed EGF-induced expression of PD-L1 mRNA and protein. In addition, inhibition of pyruvate kinase M2 (PKM2) also significantly inhibited EGF-induced PD-L1 expression and H3-Thr11 phosphorylation (171, 172). Studies have shown that inhibiting poly (ADP-ribose) polymerase-1 (PARP-1) activity can enhance p-glycogen synthase kinase 3 beta (p-GSK3β) up-regulate PD-L1 expression, and inhibit T cell infiltration (173).

#### 3.5.2 Acetylation regulation

Myocyte enhancer factor 2D (MEF2D) bound to the promoter region of the PD-L1 gene (which encodes PD-L1) and activated its transcription. Over-expression of p300 or knockdown of sirtuin 7 (SIRT7) in HCC cells promoted acetylation of MEF2D and enhanced its binding to the PD-L1 promoter region. When exposed to IFN-γ, p300 acetylated MEF2D so that it bound to the PD-L1 gene promoter and upregulated PD-L1 expression. SIRT7 also reduced the acetylation of MEF2D and the expression of PD-L1 in HCC cells without exposure to IFN-γ (174).

#### 3.5.3 Ubiquitination regulation

Ubiquitination and deubiquitination were key post-translational modifications of metabolic enzymes and contributed to the occurrence and development of various cancers, including liver cancer (175). Studies have shown that PR domain zinc finger protein 1 (PRDM1) enhances transcription of USP22 and reduces degradation of SPI1 protein through deubiquitination, thereby enhancing transcription of PD-L1 (176). GOLM1 promoted COP9 signaller-5 mediated PD-L1 deubiquitination in HCC cells and increased PD-L1 transport to exosomes by inhibiting the expression of Rab27b (177).

### 3.6 Other factors of PD-L1 elevation

#### 3.6.1 Hepatitis B virus

TME for HBV-related HCC was more immunosuppressive than in a virus-free microenvironment, and HBV- related HCC was characterized by faster progression and poorer prognosis. Studies have shown that hepatitis B x protein (HBx) plays an important role in the development of HBV-related HCC. HBx promoted cell proliferation and PD-L1 expression in tumor tissues by up-regulating the expression of S100A4 (178). In addition, studies have shown that the expression of PD-L1 in tumor tissues of HCC patients with positive pre-S2 mutations is increased (179). Clinical studies have shown an acceptable safety profile in HBV-related HCC patients. However, the anti-viral activity of PD-1/PD-L1 blockers could not be determined due to the standard anti-viral therapy performed in clinical trials. In general, except for a significantly lower disease control rate (DCR) in HBV-infected HCC patients, the objective response rate (ORR) of anti-PD-1/PD-L1 did not differ significantly between virus-positive and virus-negative patients (180). The presence of PD-L1 and PD-L2 led to suppression of the immune response, which promoted viral persistence and carcinogenesis. In addition, the expressions of PD-1, PD-L1 and PD-L2 in HCC were significantly higher than those in hepatitis, and were correlated with HCC stage and the number of infiltrating lymphocytes (181).

#### 3.6.2 Exosome

HCC cells could release exosomes containing PCED1B-AS1, which enhanced the expression of PD-Ls in HCC cells, while inhibiting the expression of T cells and macrophages (150). GOLM1 promoted the stabilization of PD-L1 and promoted the transport of PD-L1 to TAMs via exosomes, resulting in higher PD-L1 expression on TAMs than HCC cells and inducing CD8^+^ T cell inhibition. Zoledronic acid (ZA) combined with anti-PD-L1 reduced PD-L1^+^ TAM infiltration and improved CD8^+^ T cell inhibition (177). HCC cells released exosome-containing PCED1B-AS1, which enhanced the expression of PD-Ls in recipient HCC cells and inhibited receptor T cells and macrophages. PCED1B-AS1 induced the expression and function enhancement of PD-Ls through sponging hsa-miR-194-5p in HCC cells (182).

#### 3.6.3 Transarterial chemoembolization

Clinical studies have found that the expression of PD-L1 in HCC pretreated by transarterial chemoembolization (TACE) is significantly higher than that of HCC without TACE (183). Further studies have shown that rat hepatic artery embolization (HAE) can promote the expression of PD-L1 through HIF-1α (184). After TACE treatment of HCC, both the number and function of CD8^+^ T cells were impaired, while the number of TREM2^+^ TAMs was increased, which was associated with a poorer prognosis (185). TREM2^+^ TAMs produced more Galectin-1 than TREM2^-^ TAMs. Galectin-1 promoted the over-expression of PD-L1 in vascular endothelial cells and inhibited CD8^+^T cell recruitment. TREM2 deficiency also increased the infiltration of CD8^+^ T cells and inhibited the growth of HCC *in vivo* (185).

#### 3.6.4 Aflatoxin

Aflatoxins in the diet is an important risk factor for HCC (186). Aflatoxin-related HCC tissues contained high levels of potential mutation-associated neoantigens, as well as many infiltrating lymphocytes and tumor cells expressing PD-L1. In addition to the mutation of tumor protein p53 (TP53) reported in previous studies, studies also have found that there are frequent mutations of adhesion G protein-coupled receptor B1 gene (ADGRB1). ADGRB1 mutation was closely associated with increased angiogenesis and PD-L1 expression in HCC tissues (187). The expression of aryl hydrocarbon receptor (AHR) and PD-L1 was increased in HCC patients associated with aflatoxin B1 (AFB1), and anti-PD-L1 showed greater efficacy on hepatoma xenografts derived from AHR ectopic expression cells (186).

#### 3.6.5 Listeria HCC vaccine

Lmdd-MPFG promoted the expression of PD-L1 in HCC cells, resensitizing local tumor T cells in response to anti-PD-1 immunotherapy (188). Mechanistically, Lmdd-MPFG vaccine activated the NF-κB pathway in TAMs through toll like receptor 2 (TLR2) and myeloid differentiation primary response 88 (MyD88) pathways. SQSTM1 (sequestosome 1) was recruited to activate the autophagy pathway, tilting TAMs from M2-polarized TAMs to M1-polarized TAMs (188).

#### 3.6.6 Autophagy

Autophagy plays a dual role in many types of cancer, such as HCC (189). In HCC, high expression of autophagy marker mRNA was associated with poor clinical status. Increased expression of LC3 in HCC cell lines promoted tumor growth. In specific Tumor types, PD-1 or PD-L1 in tumor intrinsics was associated with higher levels of autophagy. Over-expression of PD-1 or PD-L1 increased autophagy in tumor cells through autophagy-related protein 13 (ATG13) interactions (190).

All in all, factors affecting the expression of PD-L1 in tumor cells are numerous and complex. We need to pay attention to the following key points: firstly, to find key targets regulating PD-L1 and the biological or physical factors that can affect PD-L1. Secondly, in the process of liver cancer immunotherapy, mutations in PD-L1 related regulatory genes or proteins should not be ignored, and specific inhibitors combined with ICIs should eventually be targeted for clinical or preclinical studies.

## 4 Natural products target the expression of PD-L1

Natural products play an important role in inhibiting the expression of PD-L1 in liver cancer. Most of the natural products are derived from herbs in traditional medicine, which can be used as drugs or supplements (191). In our previous study, we found that dihydroartemisinin (DHA) can not only reduce PD-L1, sensitization chemotherapy and immunotherapy (108), but also by decreasing the expression of PD-1 in CD8^+^ and CD4^+^ T cells (108). This also raises our concerns about natural products. Based on the current understanding of PD-L1, we summarized natural products that have pharmacological effects associated with PD-L1 (**Table 3**).

**Table 3:** Natural products target the expression of PD-L1 in HCC.

Name	Regulatory mechanism	Dose	Cell line/Experiment	References
Astragalus polysaccharide	Inhibits PD-L1-mediated immunosuppression via miR-133a-3p	0.1,0.5 and 1 mg/mL	SMMC-7721 and Huh-7 cells	(192)
Cantharidin	Reduces the expression of PD-L1	0.25,0.5 and 1 mg/kg	Male BALB/c mice	(193)
Chrysin	Inhibits the expression of PD-L1 by blocking the JAK/STAT3 and NF-κB pathways	1,5,10,20,40 μM and 30,120 mg/kg	HepG2 cells and male BALB/c mice	(194)
Pentamethylquercetin	Down-regulates PD-L1 expression via IFN-γ signaling pathway	1,3,10 μM and 5,10,20 mg/kg	HepG2 cells, 3T3-L1 preadipocytes and male KM mice	(195)
Quercetin	Reduces the expression of PD-L1	25,50,100 µM and 25,50,100 mg/kg	HepG2 cells and male BALB/c mice	(196)
Curcumol	Reduces the expression of PD-L1 through crosstalk between HIF-1α and p-STAT3 (T705) signaling pathways	30 μM and 3,30 mg/kg	Hep3B cells and female athymic BALB/c nude mice	(197)
Curcumin	Reduces the expression of PD-L1	10,20,30,40 µM and 10mg/kg	Hep3B, CSQT-2 cells and BALB/c female nude mice	(198)
Curcumin	Reduces the expression of PD-L1	200 mg/kg	Male BALB/c mice	(199)
Dihydroartemisinin	Reduces the expression of PD-L1 and increases CD8^+^ T cell infiltration	21.5 µM and 25 mg/kg	HepG2215, shYAP1-HepG2215 cells and C57BL/6 mice	(108)

### 4.1 Astragalus polysaccharide

Astragalus polysaccharide (APS) was one of the main bioactive ingredients extracted from Astragalus membranaceus. Studies have shown that astragalus polysaccharide has anti-tumor, anti-inflammatory, antioxidant and immune-regulating effects (200). APS dose-dependent inhibited HCC growth, IFN-γ-induced PD-L1 expression and reduced PD-L1-mediated immunosuppression of HCC cells. APS attenuated PD-L1 mediated immunosuppression in HCC cells via miR-133a-3p. In addition, miR-133a-3p targeted Moesin (MSN), which inhibited the anti-tumor effects of APS by maintaining the stability of PD-L1. In addition, APS attenuated PD-L1 mediated immunosuppression through the miR-133a-3p/MSN axis and played a vital in role anti-tumor. These results suggested that APS might be an effective drug in the treatment of HCC (192).

### 4.2 Cantharidin

Cantharidin was an insect-derived terpenoid produced by male blister beetles. Cantharidin had anti-cancer properties due to its ability to induce cell cycle arrest, DNA damage and apoptosis (201). Cantharidin inhibited the growth of HCC, increased the proportion of CD4^+^/CD8^+^T cells and B cells, and decreased the proportion of Tregs cells. In addition, it significantly reduced the expression of inflammatory factors and immune checkpoint genes PD-1/PD-L1 (193).

### 4.3 Chrysin

Chrysin was a natural flavonoid found in propolis, honey, and a variety of plants. Chrysin was a phytoestrogens that could act as ligands of the endoplasmic reticulum and has anti-inflammatory, anti-viral and cancer effects (202, 203). Chrysin significantly inhibited the overexpression of PD-L1 and increased the proportion of CD4/CD8-positive T cells by blocking the JAK/STAT3 and NF-κB pathways, inhibiting the growth of liver cancer cells both *in vivo* and *in vitro* (194).

### 4.4 Pentamethylquercetin

Pentamethylquercetin was a natural polymethoxyflavonoid with beneficial effects such as anti-tumor, anti-obesity and heart protection (204). In H22 tumor tissue of obese mice, the expression of PD-L1 was significantly increased, and the expression of ki67 was increased, while the number of CD8^+^ T cells was significantly decreased. Pentamethylquercetin down-regulated adipose-cell-induced PD-L1 expression via the IFN-γ signaling pathway, at least partially inhibiting HCC progression in obese mice (195).

### 4.5 Quercetin

Quercetin, a member of the flavonoid family, was one of the most important anti-oxidants and was widely distributed in fruits and vegetables. Quercetin had anti-oxidant, anti-inflammatory, and immunomodulatory effects and had received a lot of attention in recent years for its anti-cancer effects in a variety of cancers (205). Quercetin significantly inhibited the proliferation, migration and invasion of HCC cells *in vitro*. The levels of granulocyte-macrophage and granulocyte colony-stimulating factor (GM-CSF and G-CSF) and PD-L1 were decreased. Quercetin increased the proportion of CD86^+^ cells and decreased the proportion of CD206^+^ cells, promoting the polarization of M1 macrophages. It also increased LC3 I/II expression and down-regulated p62 expression through NF-κB pathway, promoting autophagy (196).

### 4.6 Curcumol

Curcumol was a sesquiterpenoid compound derived from *Rhizoma Curcumae* (206), which had anti-tumor, anti-inflammatory, anti-oxidant, and anti-bacterial activities (207). Studies have shown that there is crosstalk between STAT3 and HIF-1α pathways, which synergistically regulate PD-L1 activation. Curcumol inhibited PD-L1 expression in liver cancer through crosstalk between HIF-1α and p-STAT3 (T705) signaling pathways, restoring cytotoxic T cell activity and the ability to kill tumor cells (197).

### 4.7 Curcumin

Curcumin was a natural polyphenol phytochemical derived from turmeric, which had anti-oxidant, anti-inflammatory and anti-cancer properties (208). The PD-1/PD-L1 signaling pathway promoted the differentiation of Tregs through PD-L1. Curcumin in combination with TG synergistically inhibited the expression of PD-L1 and NF-κB proteins by reducing the expression of Tregs, thus inhibiting the growth of liver cancer (198). Curcumin reduced the histone acetylation of P300-induced thrombin mediated TGF-β1 promoter region, reduced the expression of PD-L1 on the surface of tumor cells or HCC cells, impeding the proliferation of tumor cells. Curcumin also increased the activation rate of lymphocytes and the expression of immune function factors, and finally delayed immune escape (199).

### 4.8 Dihydroartemisinin

DHA, a derivative of artemisinin, had anti-oxidant, anti-malaria, anti-inflammatory and anti-cancer functions (209). Our previous study found that YAP1 knockdown inhibited the expression of PD-L1, which was related to the JAK1/STAT1, 3 pathways. DHA inhibited YAP1 expression and broke immune evasion in liver cancer niche, which was manifested by decreased PD-L1 level and increased CD8^+^ T cell infiltration in liver cancer cells. In addition, DHA combined with anti-PD-1 treatment promoted CD4^+^ T cell infiltration in spleen and CD8^+^ T cell infiltration in tumor tissue (108).

These studies suggested that natural products could target the expression of PD-L1 in liver cancer and might play an important role in the treatment of liver cancer. The anti-tumor research of natural products has become a research hotspot in recent years due to its wide available resources and certain drug-forming properties (210). Therefore, the discovery of excellent PD-L1 inhibitors from natural products has created convenience for us. But the study of natural products also has limitations. Many studies on the effects of natural products on PD-L1 in liver cancer are still in the preclinical stage, and more clinical data are needed to support the safety and efficacy of natural products. In addition, the low solubility of many natural products, such as dihydroartemisinin and curcumin leads to low bioavailability. Although natural products as chemotherapy and radiotherapy sensitizers are still some time in the clinic. However, due to its good biological activity and diverse structure, it is the best object to study the mechanism of chemotherapy and radiotherapy sensitization. However, the current research is not enough, from the type and quantity of natural product studies.

## 5 Combination therapy with immune checkpoint inhibitors

Meta-analysis and subgroup analysis were performed to evaluate the benefit of PD-1/PD-L1 inhibitors in combination with advanced HCC patients. A total of 29 studies with 5396 patients were included. ICIs’ combination therapy had higher ORR (26% vs 15%) and DCR (73% vs 55%), longer PFS (5.5 vs 3.1 months) and OS (15.9 vs 12.6 months). PD-1/PD-L1 inhibitors plus anti-VEGF agents had an advantage in DCR (0.80 vs 0.48, meta-regression = - 0.32, P < 0.001), but an equal ORR (0.29 vs 0.26) compared to dual ICIs. The total OS in dua-ICIs were 16.5 months (95% CI 14.2-18.7), yet not reached in the major studies of ICI plus anti-VEGF (211). There are relatively few clinical systematic studies of ICIs combination therapy, and network meta-analyses would be particularly useful. Because there are often multiple drugs available for second-line treatment, direct head-to-head comparative trials may be lacking. By comparing existing treatments with direct and indirect evidence, clinicians can gain a broader understanding of the relative efficacy and safety of these treatments. This may include looking at OS, PFS, RR, and adverse events for different treatments. Such analyses are critical to inform clinical decision-making, establish guidelines, and identify areas where further research is needed to improve outcomes for HCC patients who require second-line treatment. In addition, we reviewed some preclinical ICIs’ combination therapy in order to provide ideas for clinical treatment.

Our previous study found that anti-PD-1 reduced the expression of PD-L1 in HCC(108). ICIs has shown a durable anti-tumor response in patients with advanced HCC, but resistance to ICIs remains in most cancer patients (212). Circulating PD-L1 could be used as an independent predictor of OS and tumor recurrence survival after cryoablation in HCC patients (213). Therefore, the most effective strategy for the treatment of advanced liver cancer might be to combine ICIs with other methods for the treatment of liver cancer, for example, the combination of ICIs with other conventional ablation therapy (such as Radiofrequency ablation (RFA) or cryoablation) will be the most promising method for the treatment of HCC. However, for unresectable advanced HCC, it was more appropriate to look for other combination strategies, such as combinations with kinase inhibitors, histone deacetylase inhibitors, and anti-viral drugs, as well as dual inhibition of two immune checkpoint molecules (214).

Studies have shown that the combination of anti-angiogenic therapy and anti-PD-1. Lenvatinib inhibited the expression of PD-L1 on human umbilical vein endothelial cells. The combined treatment of lenvatinib and anti-PD-1 also led to the formation of long-term immune memory, while synergistically regulating TME and enhancing the cytotoxicity of T cells (91). Regorafenib and PD-1 inhibitors were sequentially treated in one HCC patient with HBV-induced cirrhosis with lung metastasis, with no disease progression and mild side effects (215). However, the combination of cabozantinib with the anti-PD-L1 did not show any other therapeutic benefit in the mouse HCC model tested (216). The combination with lenvatinib was twice as effective as pembrolizumab alone, promising a median OS rate of 20 months, but at the cost of increased toxicity (217). In liver cancer, the combination of cabozantinib and nivolumab showed a significant increase in response rate, extending survival, but at the cost of more frequent and more severe toxicity (218). In mouse HCC models, the combination of ICIs (anti-CTLA-4 and anti-PD-1) with histone deacetylase inhibitor Belinostat induced early upregulation of PD-L1 on tumor antigen presenting cells and late expression of PD-1 on tumor infiltrating effector T cells. The applicability of anti-PD-1 was demonstrated (219).

Atezolizumab combined with the anti-viral 2,5-dimethylcelecoxib (DMC) in ICIs increased the level of tumor-infiltrating CD8^+^ T cells. In addition, atezolizumab promoted the ubiquitination degradation of HBx and induced PD-L1 protein in HCC cells by activating 5’-adenosine monophosphate to activate the protein kinase pathway, which plays a more significant anti-tumor effect (91).

Double ICIs had a synergistic effect and a higher response rate and better efficacy compared to monotherapy (214). CTLA-4 and PD-1 had similar mechanisms in terms of tumor tolerance. As a result, most patients with advanced HCC using anti-PD-1/PD-L1 did not achieve lasting control, and the combination with anti-CTLA-4 improved treatment effectiveness (220). Based on current evidence, several first and second line phase 3 randomized trials in HCC patients have been initiated, although it will be several years before mature survival data are available.

## 6 Conclusion and future perspectives

In summary, PD-L1 is expressed on a variety of cells in the immune microenvironment of HCC, including macrophages, monocytes and tumor cells. In addition, in the tumor microenvironment, different cells will interact with each other to further promote or inhibit the expression of PD-L1, which also increases the difficulty of HCC immunotherapy. The technology of spatial omics and advanced computational methods have been developed rapidly. Therefore, we should fully consider the spatial diversity of PD-L1. A study has also shown that the spatial interaction between PD-1/PD-L1 and IDO-1/HLA-DR is closely related to anti-PD-1 clinical response (221). Spatial quantification of the tumor immune microenvironment may have better prognostic ability than existing biomarkers, and further development and application of spatial omics may promote a new revolution in the tumor immune microenvironment ecosystem (222).

In addition, in addition to studying the role of PD-L1 in immunotherapy, it has also been found that PD-L1 can also act as a proto-oncogene to regulate the conduction of other signaling pathways of tumor cells and directly promote tumor growth (223) and metastasis (224). In particular, the research on PD-L1 and tumor metastasis has become a hot topic. For example, in colorectal cancer liver metastasis, PD-L1 is still highly expressed in tumors at the site of metastasis. Moreover, the application of PD-1 inhibitors is still effective (225). In addition, there is a unique immune landscape in liver metastasis of rectal cancer, and highly metabolically activated MRC1^+^ CCL18^+^ M2-like macrophages are found at the site of metastasis (226). The expression of PD-L1 in intestinal metastasis of lung cancer also has similar characteristics (227). Metastasis is also the most serious complication in the process of tumor treatment. The influence of PD-L1 expression in metastatic cancer cells on the fragile microenvironment at the site of metastasis is still unknown. Therefore, the study of PD-L1 in tumor metastasis will also become a hot spot in the future, and corresponding technical means will continue to appear (228). Therefore, studying the regulation of PD-L1 expression in HCC is beneficial to the treatment of HCC in many ways.

Furthermore, the regulation of PD-L1 expression is regulated by many factors, including transcriptional, post-transcriptional, translational and post-translational multifaceted regulation, and cross-regulation exists among different levels and signaling pathways, and the mechanism is complex. Therefore, it is increasingly necessary for us to find the key signaling pathways regulating PD-L1. The positive expression of PD-L1 in tumor tissue is regarded as an indicator of the application of ICIs, but PD-L1 positive patients are not fully effective in ICIs therapy, on the contrary, PD-L1 negative patients can also benefit from ICIs therapy. Therefore, in the process of research, we should pay more attention to what kind of cells in the tumor microenvironment express PD-L1, which affects the tumor immunotherapy and the precise application of drugs.

In addition, we still need to deeply understand how PD-L1 plays an immunosuppressive role in liver cancer, and some studies have also brought us some inspiration. PD-1 on the surface of CD8^+^T cells inhibits T cell glycolysis by inhibiting PI3K/Akt/mTOR signaling pathway. On the one hand, PD-L1 on the surface of tumor cells can bind to PD-1 on the surface of CD8^+^T cells to inhibit T cell function. On the other hand, it can promote the translation of glycolytic enzyme GLUT1 by promoting the Akt/mTOR signaling pathway of tumor cells in the tumor microenvironment. Glucose deprivation by tumor cells affects the glucose demand of T cells. On the one hand, in the tumor microenvironment, anti-PD-L1 treatment can inhibit the Akt/mTOR pathway to reduce glucose consumption and also enhance the ability of CD8^+^ T cells to compete for glucose. On the other hand, it also alleviates the negative effect of PD-1 on CD8^+^ T cells, restores the glycolysis function of T cells, and increases the production of cytokine IFN-γ (153). Tumor cells in liver cancer glycolysis changes profoundly affect the immune cells in the tumor microenvironment (229). Understanding the relationship between PD-L1 and immunosuppression from the perspective of glycolysis may be only one aspect, and more problems need to be explored.

Therefore, the therapeutic strategy of combining ICIs has expanded a new space for the application of ICIs, and has also shown a very obvious therapeutic effect. This will ultimately lead to more choices for patients and more benefits for longer patient survival.

## Author contributions

LH: Investigation, Software, Writing – original draft. SL: Conceptualization, Data curation, Writing – original draft. JD: Writing – original draft. NL: Writing – original draft. FY: Writing – original draft. ZJ: Writing – original draft. JZ: Writing – original draft. XS: Writing – original draft. XH: Funding acquisition, Visualization, Writing – original draft.
